# DFT study of alkaline earth metals NaXH_3_ (X = Be, Mg, Ca, Sr) for hydrogen storage capacity

**DOI:** 10.1039/d4ra05327c

**Published:** 2025-01-02

**Authors:** Danial Tufail, Umair Ahmed, Mazhar Haleem, Bin Amin, Muhammad Shafiq

**Affiliations:** a Department of Physics, Abbottabad University of Science & Technology Abbottabad 22020 Pakistan shafiqdurraniuom@gmail.com +92 346 8006800

## Abstract

The potential application of materials referred to as perovskite hydrides in hydrogen storage – a crucial element of renewable energy systems – has sparked a great deal of interest. We use density functional theory (DFT) to investigate the structural, formation energy, hydrogen storage, electronics, thermoelectric and elastic properties of NaXH_3_ (X = Be, Mg, Ca, and Sr) hydrides. The band gap is calculated using WC-GGA and WC-GGA+mBJ potentials. WC-GGA+mBJ potentials show improvement in band gap values. The thermoelectric properties of these compound are studied using post-DFT Boltzmann's techniques. The elastic constants and mechanical properties of the hydrides, such as their Shear modulus, Young’s modulus, Pugh ratio, Poisson ratios, anisotropic index and micro-hardness, are also calculated. Our findings show that all materials are mechanically stable and satisfy the Born criteria. The higher gravimetric ratios of all materials are good enough for storing hydrogen and can be used for advanced future applications. Furthermore, NaSrH_3_ is the perfect candidate for thermoelectric applications due to its higher power factor and figures of merit *ZT* (≈ 1).

## Abstract 2

We use DFT to investigate the structural, formation energy, hydrogen storage, electronics, thermoelectric and elastic properties of NaXH_3_ (X = Be, Mg, Ca, and Sr) hydrides as potential hydrogen storage materials.

## Graphical abstract

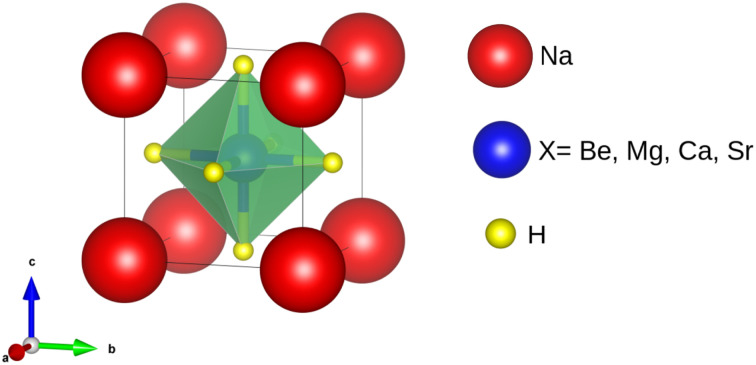

## 1 Introduction

With the rise in population, there has been a significant increase in global energy consumption. Hydrogen is considered one of the most promising energy carriers.^1^ In order to provide humanity with cutting-edge facilities, research and technology must provide solutions to the demand for the day by day energy increases.^2^ Most energy generated comes from fossil fuels, which are non-renewable and take longer to replenish or return to their starting form.^3^ As the world's energy needs increase, innovative strategies and research on sustainable energy are also updated.^4^ Although hydrogen has a few obstacles to overcome, it is undoubtedly a fantastic alternative to fossil fuels like coal, oil and natural gas.^5^ Because of environmental contamination and the use of fossil fuels, there is an increasing need for clean and economical energy sources. Renewable and electrified energy sources are being studied extensively.^6,7^ Hydrogen presents itself as an exceptional gas for use as a source of energy, but the most challenging aspect currently is storing hydrogen.^8^ For cars, computers, and mobile devices, hydrogen is a promising energy source that has the potential to displace non-renewable petroleum derivatives. Burning hydrogen can lower carbon dioxide emissions and is environmentally friendly, efficient and sustainable.^9^ Numerous materials have been investigated for the storage of hydrogen, such as complex hydrides, nanomaterials, and graphene-based materials. A high rate of hydrogen is needed for practical use in order to make it a viable alternative to fossil fuels.^10^ Researchers are working on a variety of perovskite materials that have exceptional hydrogen storage capacity. Hydrogen storage materials are made up of specific metals, compounds, and a special form of nano structured hydrides, which is made up of microscopic particles.^11^ Certain parameters must be met by the material used for energy storage, including a high volumetric and gravimetric ratio, good kinetic energy, noteworthy mechanical qualities, and the capacity to release hydrogen under normal circumstances.^12,13^ ABH_3_ perovskite is a hydride perovskite with a structure in which B is a light element that replaces one of the O-atoms in the BO_6_ octahedral, such as carbon (C), oxygen (O), or nitrogen (N). When lighter elements are substituted for oxygen, hydrogen forms more bonding sites, resulting in high storage capacity.^14,15^ They are different categories as the elements in groups 1 and 2 of the periodic table can be used to create A and B. The second class of hydrides of the perovskite type is produced by combining monovalent alkali or divalent metals A and B.^16,17^ This category consists of SrPdH_3_, LiCuH_3_, LiFeH_3_, MgFeH_3_, CaNiH_3_, MgCoH_3_, CaCoH_3_ and KCuH_3_.^18,19^ Due to their high gravimetric densities, light metal hydrides are among the most promising materials for on-board hydrogen storage.^20^ Metal hydrides are examples of functional compounds that aid in the absorption of hydrogen.^21^ Hydrogen is stored in vast amounts in the intermetallic phases of various metals through chemical bonding.^22^

NaMgH_3_ and Na_0.9_K_0.1_MgH_3_ have been experimentally synthesized using a high-energy ball milling method, which revealed that adding different concentrations of K on Na enhanced the dehydriding kinetic properties and increased the amount of hydrogen desorbed.^23^ Song *et al.*^24^ found that the gravimetric hydrogen storage capacities of NaMnH_3_, KMnH_3_, and RbMnH_3_ compounds are 3.74, 3.12, and 2.11 wt%. Li *et al.*^25^ studied the electronic structure of NaMgH_3_ and reported that it behaves as a metal, while Bouhadda *et al.*,^21^ Fornari *et al.*^26^ and Vajeeston *et al.*^27^ found theoretically that NaMgH_3_ must be a semiconductor. In this paper we reported the electronic behavior of NaMgH_3_ using a different exchange correlation potential in order to accurately treat the band structure.

The need for clean and renewable energy sources has become a serious challenge. In this regard, thermoelectric materials and hydrogen are very popular as a potentially broad, effective, and sustainable energy source. By solving the heating problems linked to storing hydrogen, thermoelectric properties can help make hydrogen a successful and sustainable energy source in the long term. Also, it has been found that thermoelectric devices can be used as hydrogen solid storage tanks.^28^ In contrast to compressed gas or liquid hydrogen, perovskite hydrides of the type NaXH_3_ (X = Be, Mg, Ca and Sr) are considered safer options for storing hydrogen. This study provides important information that could improve the efficiency and safety of hydrogen storage technologies in the future by examining the characteristics of these materials. For hydrogen storage applications we calculated the gravimetric ratio and formation energy. Furthermore, other important properties such as structural, electronic, mechanical and thermoelectric characteristics of NaXH_3_ (X = Be, Mg, Ca, Sr) hydride perovskites are also calculated and studied in this article.

## 2 Computational details

Density functional theory (DFT) and an approach known as full-potential linearized augmented plane wave plus local orbitals (FP-LAPW+lo) implemented in WEIN2k code is used in the first principles calculations of NaXH_3_ (X = Be, Mg, Ca, and Sr).^29,30^ We used WC-GGA as an accurate exchange-correlation potential in our calculations.^31^ To ascertain these properties, the WC-GGA is employed in conjunction with the modified Beck-Johnson (mBJ) approach.^32^ mBJ is one of the best density functional approaches (error of about 2%) to treat the electronic band structure of perovskites and various semiconductor materials.^33^ We use the finer, 7 × 7 × 7 and the denser 10 × 10 × 10 k-mesh to determine the structural and electronic properties. To obtain the elastic constant, the energy strain approach which is included in the WEIN2k package was applied.^34^ By subjecting the cubic lattice to deformation, only three independent elastic constants *C*_11_, *C*_12_ and *C*_44_ can be calculated. Thermoelectric characteristics including figure of merit (*ZT*), power factor (PF), electronic thermal conductivity (*κ*), electrical conductivity (*σ*), and Seebeck coefficient (*S*) were investigated utilizing WC-GGA+mBJ through the extended semi-classical BoltzTraP package.^35^ Different possible geometries are designed and visualized using VESTA, with the assist of vesta code we obtained the polyhedral structure of these perovskite materials.^36^

## 3 Results and discussion

### 3.1 Structural properties

**Fig. 1:**
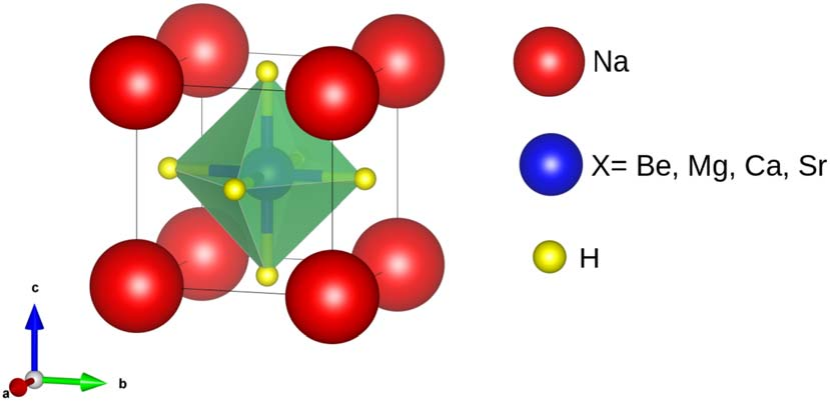
Crystal structure of NaXH_3_ (X = Be, Mg, Ca, Sr) perovskite-type hydrides.

NaXH_3_ (X = Be, Mg, Ca, Sr) hydrides have the *Pm*3̄*m* (221) space group. The unit cells of these compounds were figured out using fractional coordinates. The Na-atom is found at the corner position (0, 0, 0), the X metal atom is located at the center position (0.5, 0.5, 0.5) and the H-atom occupies the center position of the face of the octahedral sites (0.5, 0, 0), (0, 0.5, 0) and (0, 0, 0.5). In Fig. 1, the structural arrangement is presented.

Fig. 1 shows that the Na-atoms (red sphere) are cations which occupy the corner of a cube. The X-atom (blue sphere) (X = Be, Mg, Ca, Sr) also serves as a cation but with a position at the center. The face of the cube is occupied by hydrogen atoms (yellow sphere) serving as anions.

**Fig. 2:**
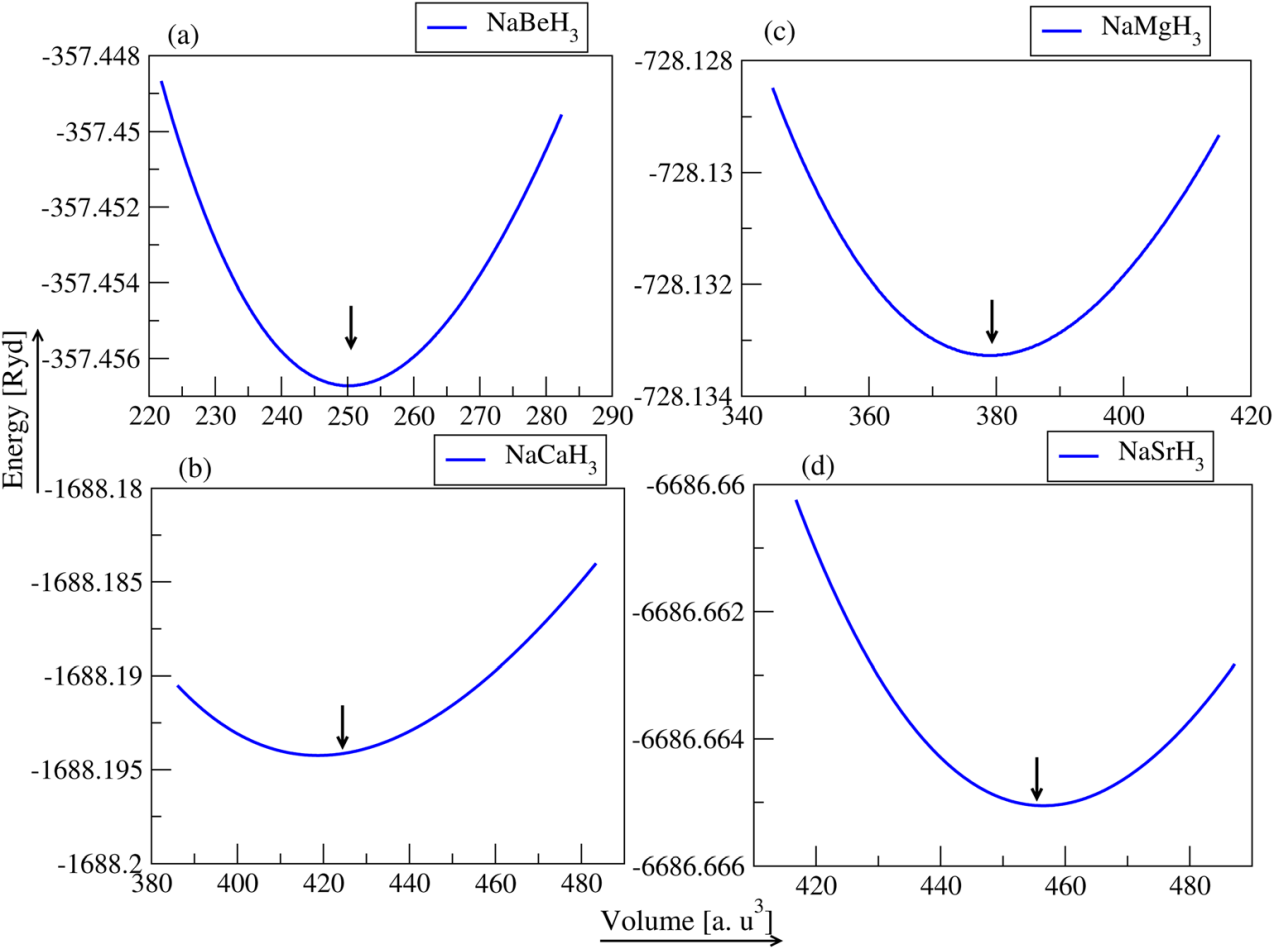
*E*–*V* curve of NaXH_3_ (X = Be, Mg, Ca, Sr), arrow shows the minimum energy point.

The structural stability of perovskite materials can be understood using energy–volume curves. The *E*–*V* curves for NaXH_3_ (X = Be, Mg, Ca, Sr) are shown in Fig. 2(a)–(d). The *E*–*V* plot for any material provides very valuable information about its mechanical structure and dynamic stability. Achieving a ground state is the first step in the stability of any physical compound that can be found by plotting an energy graph in terms of its volume.^37^ Forces were firstly calculated, using the WC-GGA function. The system was unfastened until the forces acting on the atoms were negligible. The optimized lattice constants were obtained using the Birch–Murnaghan equation of state,^38^1
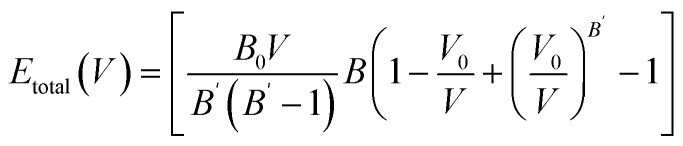
where in eqn (1)*B*_0_, *B*′, *V*_0_ and *E*(*V*) are the bulk modulus, first derivative of *B*, unit cell volume and energy at equilibrium ground states, respectively. Fig. 2 shows the evolution of the total energy as a function of the unit cell volume of cubic perovskite-type NaXH_3_. The equilibrium lattice parameter was computed also from the structural optimization, using the Birch–Murnaghan equation of state listed in Table 1. The *E*–*V* plot is generated by varying the lattice constant and calculating the minimal energy point – the lowest point of the curve where the arrow is set – which shows where the structure is dynamically stable. *E*–*V* plot provides important details regarding the properties and structural stability of the NaXH_3_ (X = Be, Mg, Ca, Sr) material. Table 1 shows that the calculated lattice constants are in agreement with other reported values. The previous studies^20–22^ on the same type of hydrides show that these materials may be used for hydrogen storage applications. When a substance absorbs hydrogen, its stability may be ascertained using the *E*–*V* curve. A material is said to be capable of efficiently storing hydrogen if its energy dramatically drops as its volume increases. This connection guarantees that the material stays stable and effective for storage while demonstrating how well it can withstand the changes that occur during hydrogen absorption, which could be employed in hydrogen storage applications.

**Table 1:** The calculated lattice constants (Å), bulk's modulus *B* (GPa), volume (Å^3^), formation energy (Δ*H*_f_) and gravimetric ratio (*C*_wt%_) of perovskite hydride NaXH_3_ (X = Be, Mg, Ca, Sr)

Compound	a (Å)		*C*_wt%_	Δ*H*_f_ (eV per atom)
	Present work	Previous work		
NaBeH_3_	3.397	3.281 (ref. 20)	8.6	−0.285
NaMgH_3_	3.879	2.009 (ref. 21)	6.0	−0.241
NaCaH_3_	3.958	—	4.5	−0.134
NaSrH_3_	4.077	—	2.6	−0.107

### 3.2 Formation energy and hydrogen storage

To compute the thermodynamical stability of NaXH_3_ (X = Be, Mg, Ca, Sr) their formation energies Δ*H*_f_ are determined using the equation:^39^

2 Δ*H*_f(NaXH_3_)_ = [*E*_total(NaXH_3_)_ − *E*_s(Na)_ − *E*_s(X)_ − 3/2(*E*_s_*H*_2_)].

**Fig. 3:**
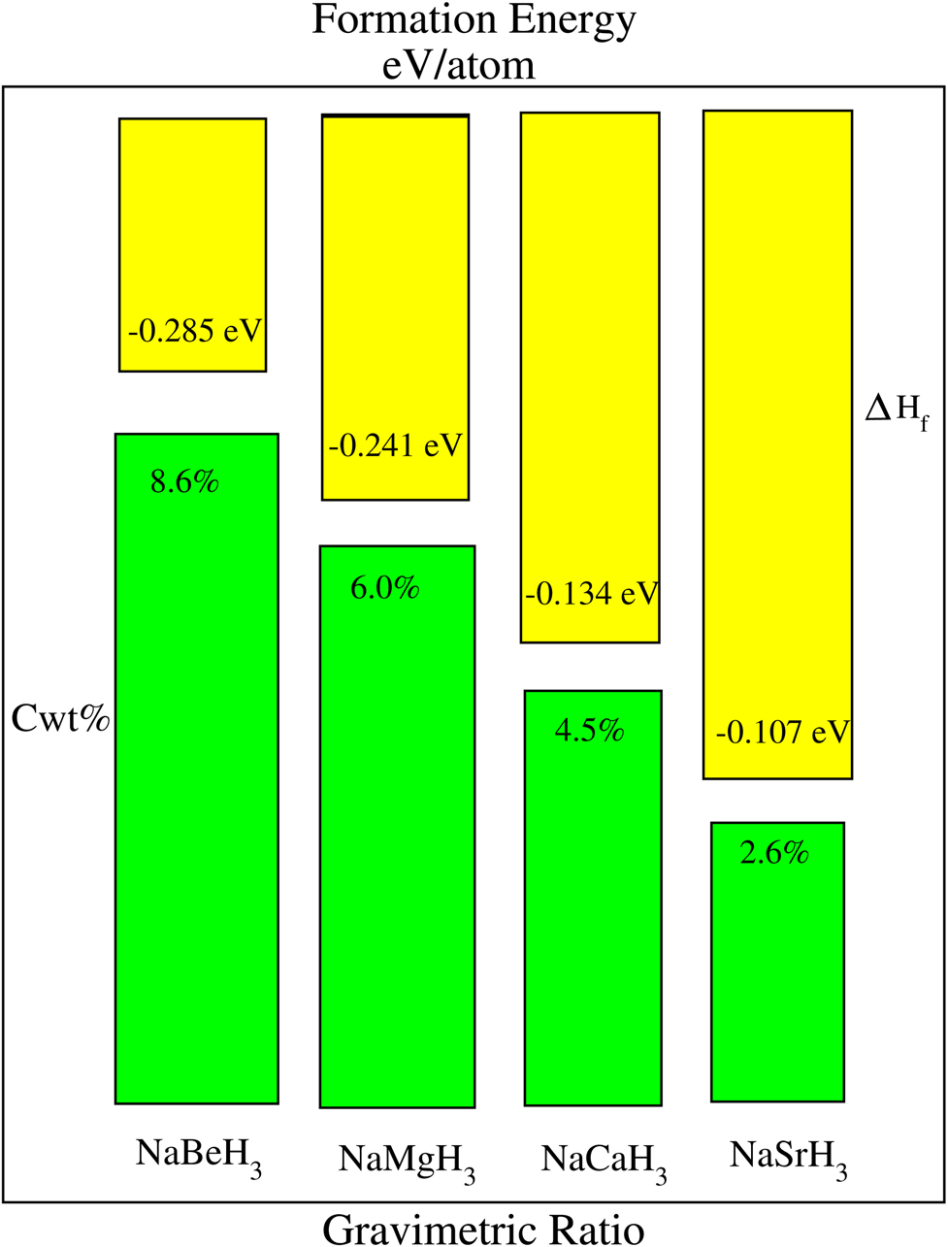
Formation energy Δ*H*_f_ (yellow bars) and gravimetric storage capacity *C*_wt%_ (green bars) of NaXH_3_ (X = Be, Mg, Ca, Sr).

In eqn (2) the individual ground state energies *E*_s(Na)_, *E*_s(X)_ and *E*_s_*H*_2_, as well as *E*_total(NaXH_3_)_, the energy of the whole compound, are used. The stability formalisms for the formation energies of NaXH_3_ perovskite materials are shown in Fig. 3. The figure shows that the calculated value of all materials have a negative formation energy (yellow bar), indicating that these materials are thermodynamically stable. The negative formation energy of NaXH_3_ is also listed in Table 1. NaBeH_3_ has the lowest formation energy (−0.285) among all the materials under consideration and is the most thermodynamically stable material along with NaCaH_3_ and NaMgH_3_.

The potential for hydrogen storage applications of NaXH_3_ (X = Be, Mg, Ca, Sr) hydride perovskites has been assessed by calculating their gravimetric storage capacity using eqn (3), the hydrogen deposited is represented by the gravimetric ratios. The algorithm utilized to assess the cwt% for gravimetric ratio is given by:^40,41^3
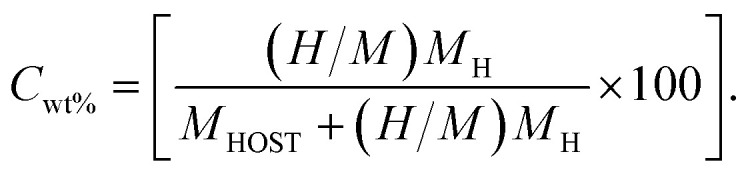


In eqn (3), *M*_H_ represents the weight of hydrogen atoms, *M*_HOST_ represents the weight of the material it's in and *H*/*M* is the ratio of hydrogen atoms to material atoms. Fig. 3 represents the weight percentage of the gravimetric capacity of perovskite hydrides NaXH_3_ (X = Be, Mg, Ca, Sr) in which NaBeH_3_ can hold the most at 8.6% and NaMgH_3_ can hold 6.0%. Also, NaCaH_3_ can store 4.5% but NaSrH_3_ can only store 2.6%. Therefore, NaBeH_3_, NaMgH_3_, and NaCaH_3_ have good gravimetric ratios. The energy–volume curve demonstrates a material's stability by displaying how its energy varies with volume. Lower energy states correspond with lower formation energies, making the material better suited for hydrogen storage. The lowest formation energy of NaBeH_3_ compounds allows hydrogen to enter and exit more easily compared to other compounds, and achieves a hydrogen gravimetric capacity of 8.6%. High formation energy can make hydrogen storage less efficient. These principles work together to determine the best materials for successful hydrogen storage applications.^42,43^

### 3.3 Electronic properties

Examining the electronic structure of compounds allows us to better comprehend their solid form. Several major characteristics of materials can be better understood thanks to such studies. Both the band structure and the total density of electronic states are important in the study of solid construction.

#### 3.3.1 Band structure

**Fig. 4:**
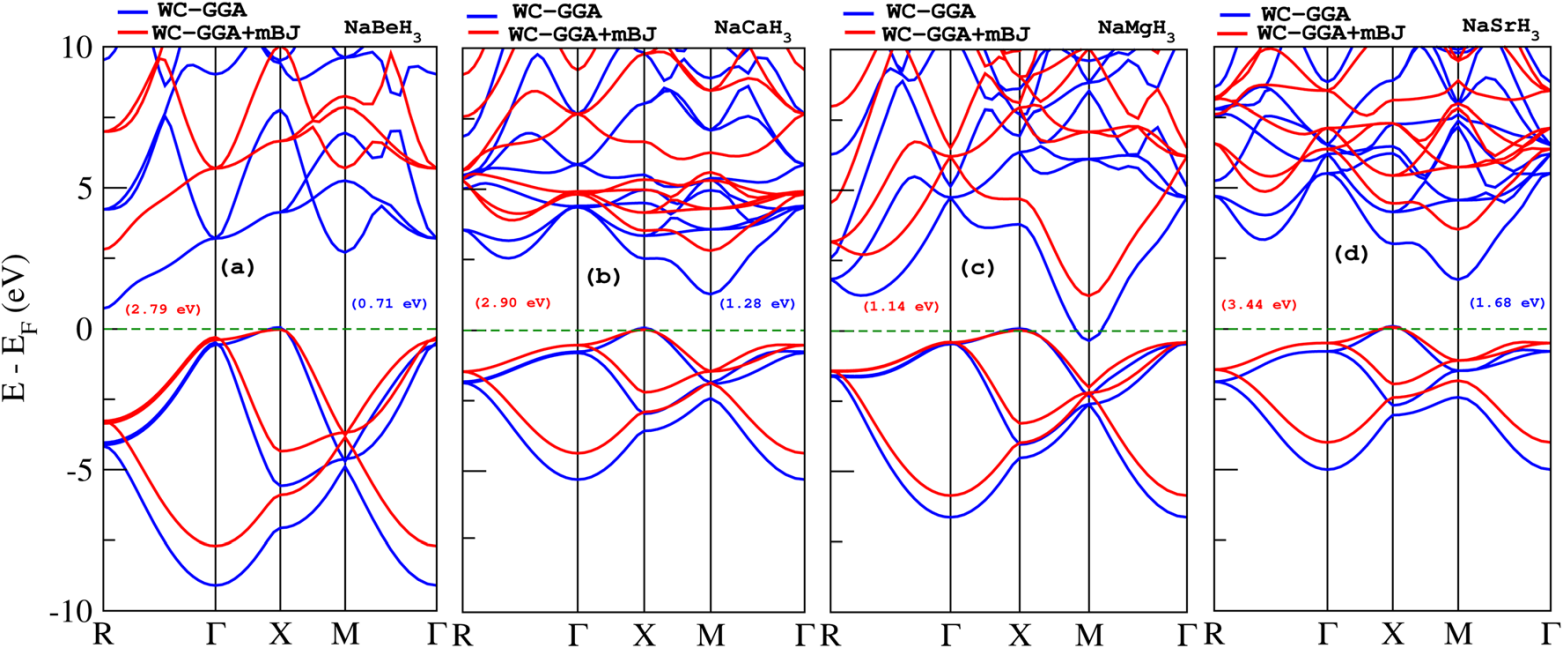
The band structure of NaXH_3_ (X = Be, Mg, Ca, Sr) with WC-GGA and WC-GGA+mBJ.

The band structure for the NaXH_3_ (X = Be, Mg, Ca, Sr) cubic phase has been calculated along the high symmetry directions in the first Brillouin zone, with WC-GGA and WC-GGA+mBJ exchange potential method. Fig. (4a–d) display the band structure of NaXH_3_. We notice that the minima and maxima of the conduction and valence band are not situated at the same point of symmetry. Therefore, these materials have an indirect band gap transition in both correlations. The material NaBeH_3_ has Γ–R and NaMgH_3_, NaCaH_3_ and NaSrH_3_ have X–M direction, which validates that our materials have semiconductor nature. The calculated band gaps for all considered materials are given in Table 2.

**Table 2:** Calculated band gap with WC-GGA and WC-GGA+mBJ NaXH_3_ (X = Be, Mg, Ca, Sr)

Compound	Present work		Other work	
	WC-GGA	WC-GGA+mBJ	WC-GGA	GGA+mBJ
NaBeH_3_	0.71 eV	2.79 eV	0.76 eV	3.1 eV (ref. 20)
NaMgH_3_	0.0 eV	1.14 eV	—	—
NaCaH_3_	1.28 eV	2.90 eV	—	—
NaSrH_3_	1.68 eV	3.44 eV	—	—

Using the WC-GGA approximation (blue lines in Fig. 4), the *E*_g_ values for NaBeH_3_, NaCaH_3_ and NaSrH_3_ compounds are found to be 0.71 eV, 1.28 eV, 1.68 eV, respectively, while NaMgH_3_ shows metallic nature using WC-GGA. Whereas the WC-GGA+mBJ approach (red lines in Fig. 4) changes the band gap nature of NaMgH_3_ material from metallic to semiconductor with a band gap value of 1.14 eV. Similarly, the improved band gap of NaBeH_3_, NaCaH_3_ and NaSrH_3_ are 2.79 eV, 2.90 eV and 3.44 eV, respectively.

The electronic band gap has a major impact on the capacity to store hydrogen. To permit ideal electron transport between the material and hydrogen from the valence to conduction band, a modest band gap that increases binding energy is required, as in our computed data for NaBeH_3_, explaining its better hydrogen intake of up to 8.6%. Additionally, such a band gap enhances electrical conductivity, which promotes the passage of hydrogen. The band gap also helps predict phase stability during the absorption and desorption of hydrogen.

#### 3.3.2 Density of states

**Fig. 5:**
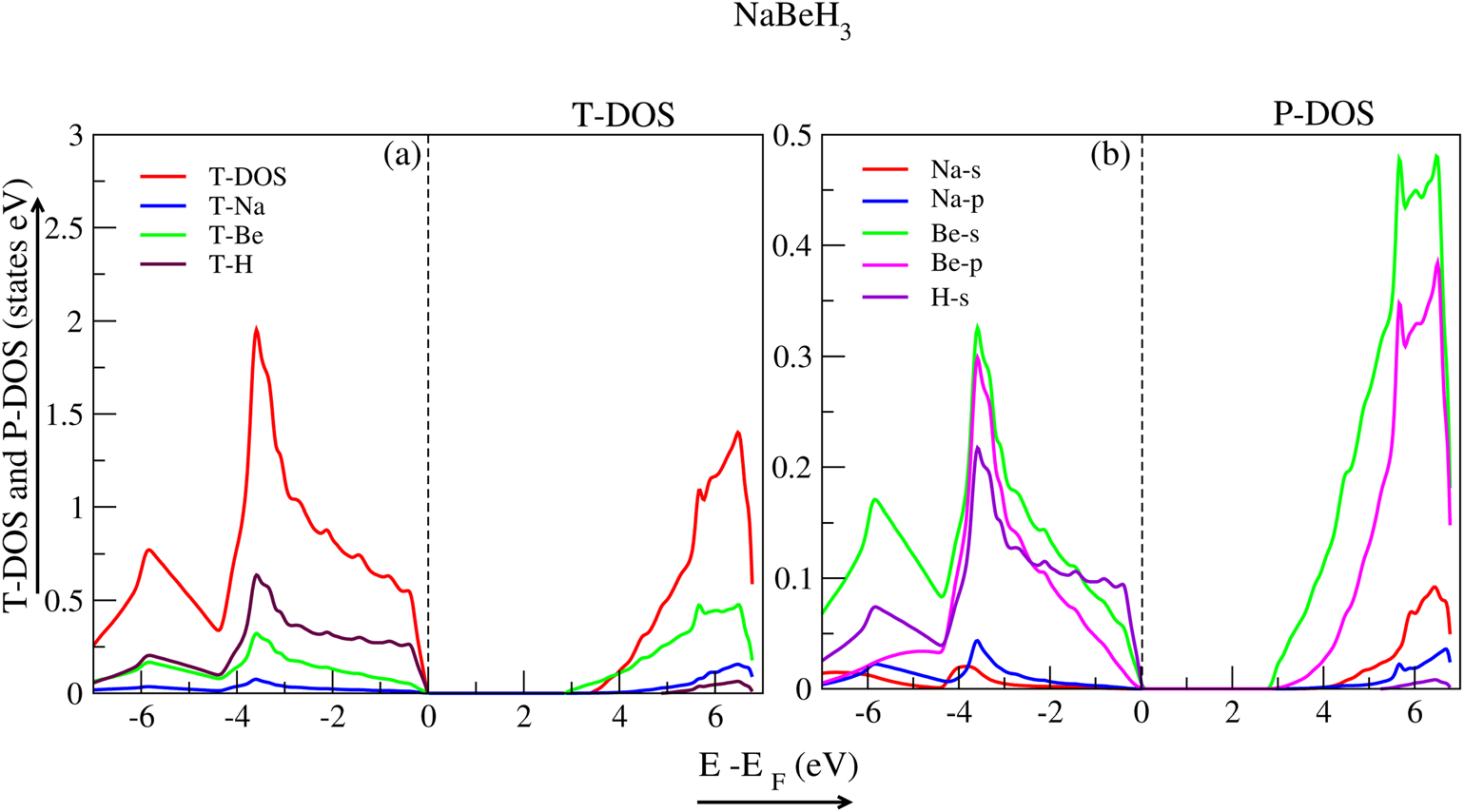
Total and partial density of states for NaBeH_3_.

**Fig. 6:**
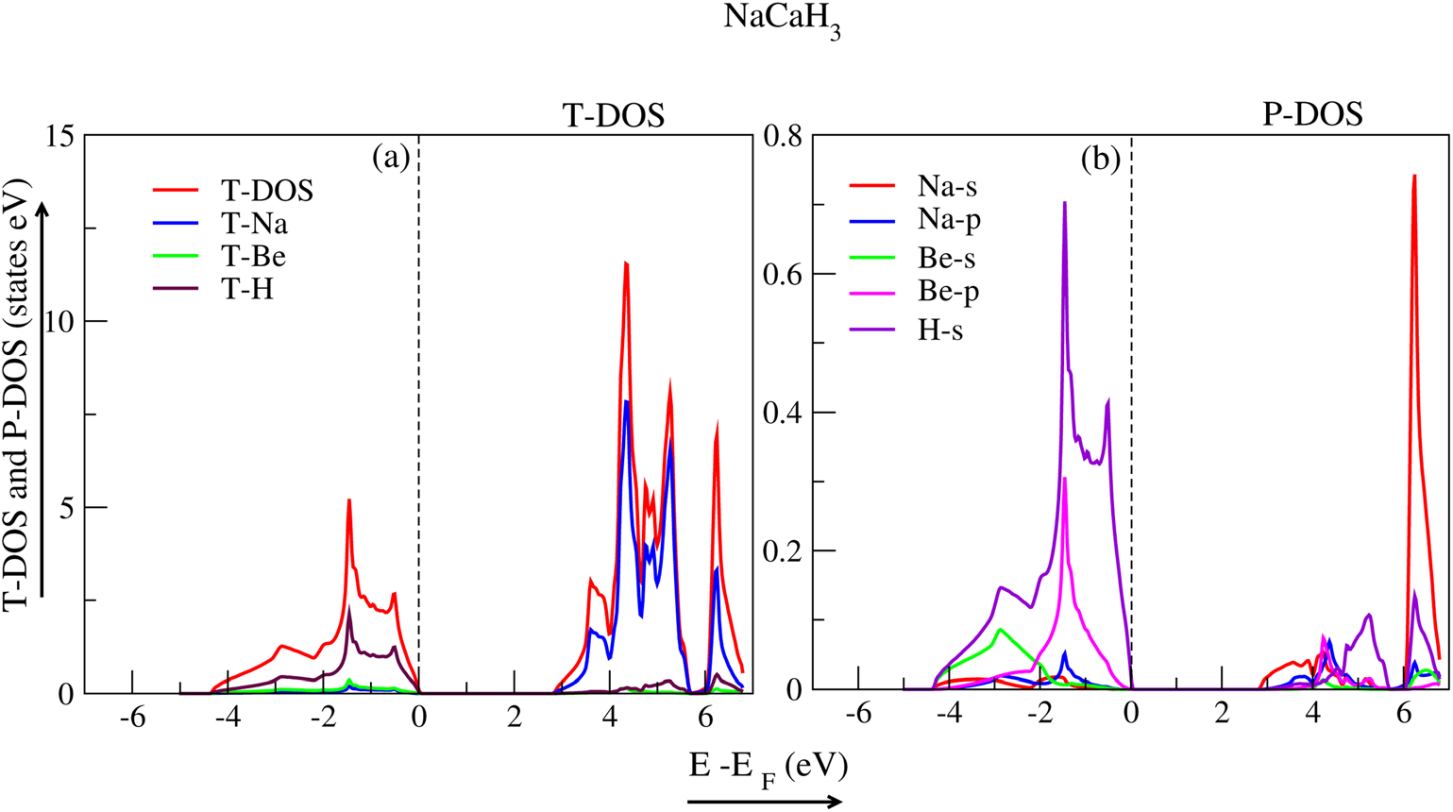
Total and partial density of states for NaCaH_3_.

**Fig. 7:**
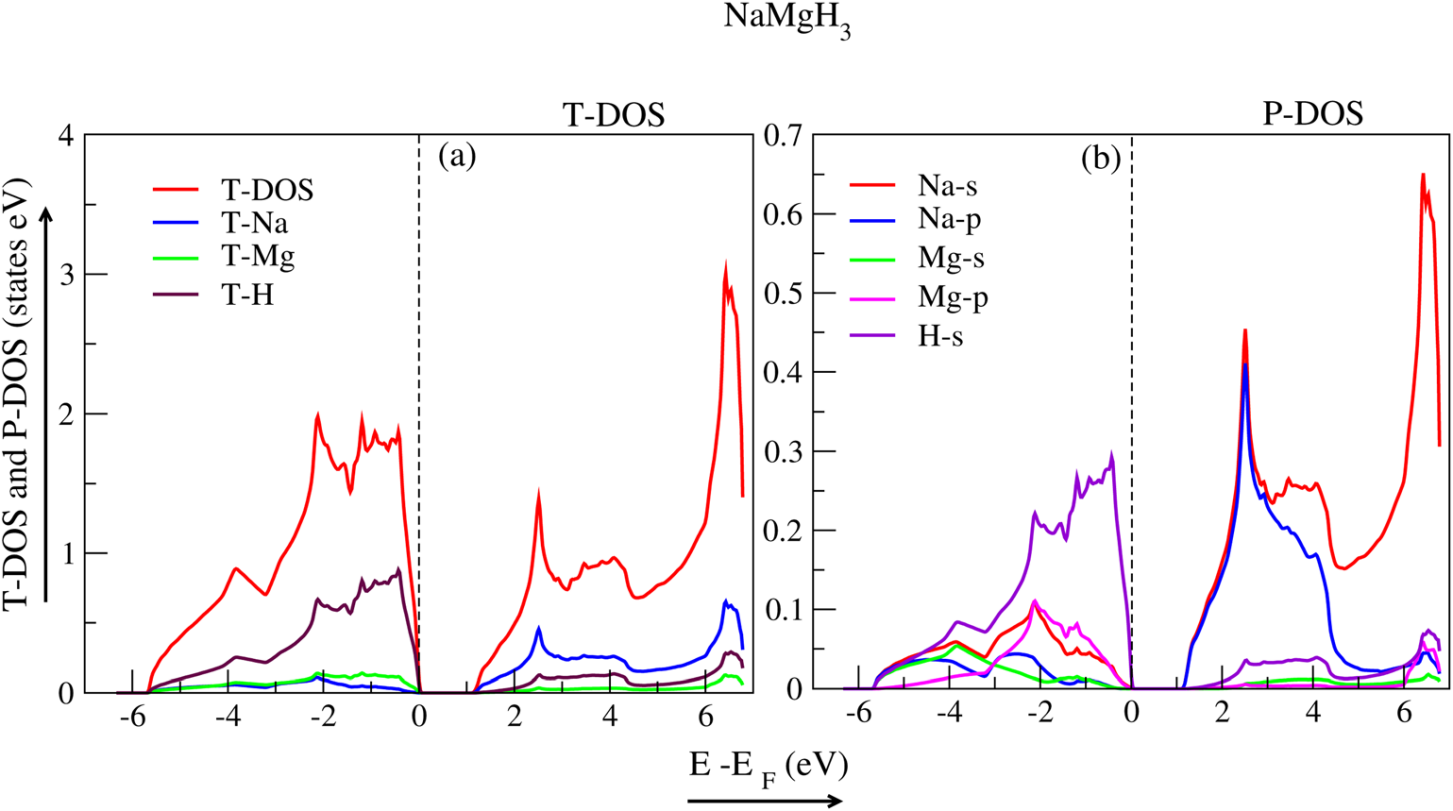
Total and partial density of states for NaMgH_3_.

**Fig. 8:**
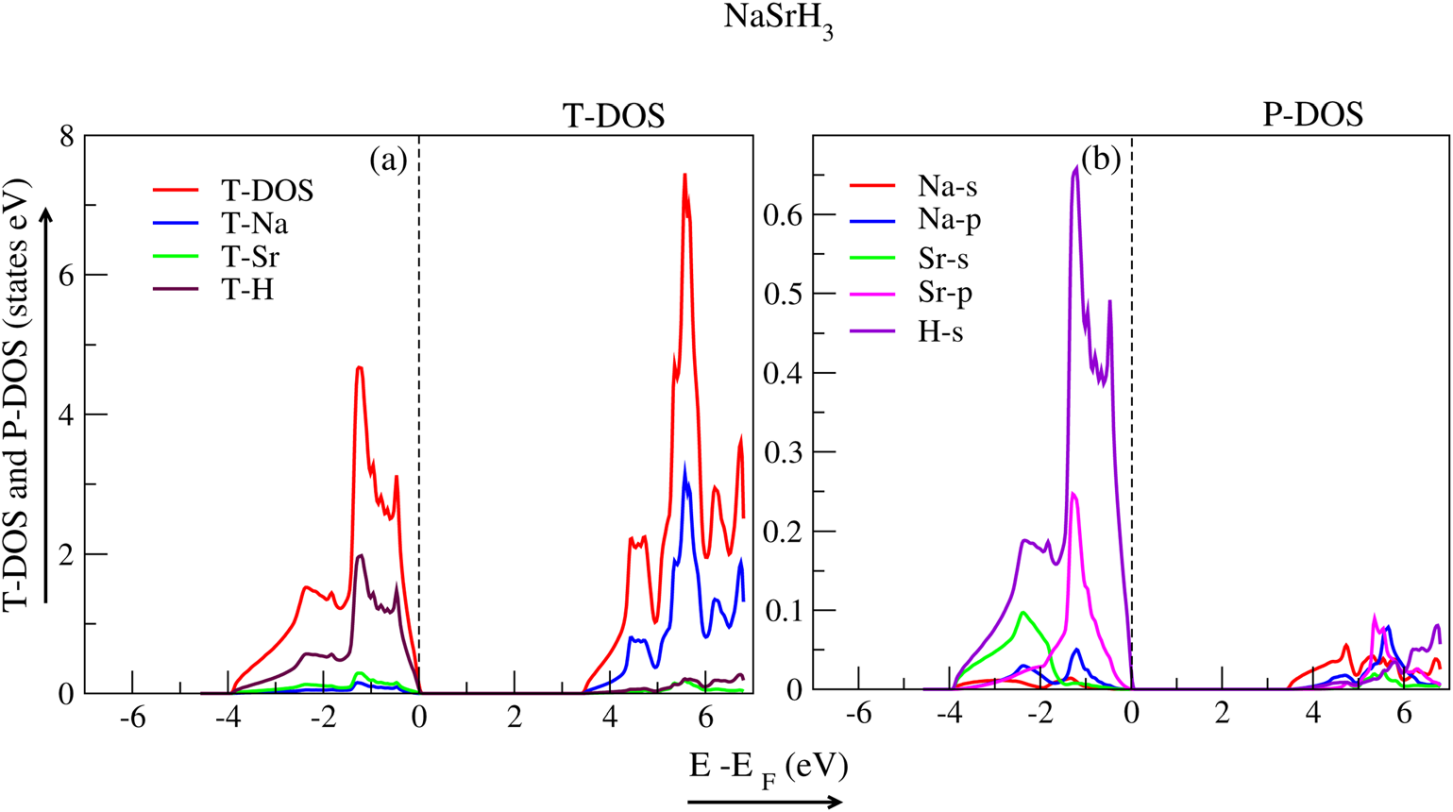
Total and partial density of states for NaSrH_3_.

Density of states (DOS) is used to scrutinize how electronic band structure is affected due to atomic exchange and relaxation distribution of energy levels. By examining DOS, one can discern if a material contributes metallic or semiconductor behaviour. The partial density of states (P-DOS) gives insight into the role of an electron's orbital in the conduction and valence band. The DOS for each material were examined using WC-GGA+mBJ. The total density of states (T-DOS) and P-DOS for the hydride perovskite NaXH_3_ materials are displayed in Fig. 5–8. Strong hybridization between hydrogen, sodium and the X atom (X= X = Be, Mg, Ca, Sr) is found, as expected. Therefore, hydrogen interaction with other atoms increases the stability of the compounds as shown in the *E*–*V* plots (Fig. 2).

Fig. 5(a) shows the T-DOS of NaBeH_3_ (red line). Fig. 5(a) reveals the primary influence of the individual atoms (Na, Be and H) in both the valence band (VB) and conduction band (CB). The P-DOS of NaBeH_3_ in Fig. 5(b) shows that the VB and CB can be attributed to the s and p-orbitals of the Be-atom, while the VB can also be attributed to the s-orbital of the H-atom. Fig. 6(a) represents the T-DOS of NaCaH_3_. The CB can be attributed to the dominant peak of the Na-atom (blue peak); the T-DOS are depicted by (red peaks). Fig. 6(b) shows the P-DOS of NaCaH_3_, where the s-orbital of the H-atom and p-orbital of the Be-atom have major influence on the VB (violet and magenta peak), while in CB the s-orbital of the Na-atom plays a vital role.

The T-DOS of NaMgH_3_ are presented in Fig. 7(a), where the significant contribution of the H-atom in the VB and Na-atom in the CB are shown with the maroon and blue peaks, respectively. The P-DOS of NaMgH_3_ is presented in Fig. 7(b), where the s-orbital of the H-atom has a major contribution to the VB, and the CB can be attributed to the s and p-orbitals of the Na-atom. Fig. 8(a) shows the T-DOS of NaSrH_3_ with major contribution of the H-atom to the VB (maroon peak), and Na-atom (blue peak) to the CB, while the red peak indicates the T-DOS. Fig. 8(b) shows that the P-DOS of NaSrH_3_ has a significant contribution from the s-orbital of the H-atom and p-orbital of the Sr-atom, and the VB is contributed by the p-orbital of Na and Sr-atom in CB.

DOS is significant for hydrogen storage because it indicates the availability of electronic states suitable for hydrogen binding. A high DOS near the Fermi level suggests that a material has more binding sites, which boosts its capacity to absorb hydrogen; in the present work NaBeH_3_ has the highest DOS. Furthermore, the DOS structure affects the energy levels associated with hydrogen interactions, which in turn affects binding energies. An optimal DOS promotes efficient charge transfer, which stabilizes hydride phases during absorption and desorption. Overall, a positive DOS contributes to improved reaction kinetics and stability, making it important for efficient hydrogen storage.^44^

### 3.4 Thermoelectric properties

Thermoelectric properties play a crucial role in heat transfer, such properties have diverse applications in storing energy effectively and providing solutions to various problems. Thermoelectric materials are especially valuable in thermoelectric devices as they can convert waste heat into usable electrical energy. By employing thermoelectric devices to harness and utilize wasted engine heat, significant cost savings can be achieved. The use of thermoelectric materials holds promise in addressing society's energy challenges. Examples of different types of thermoelectric properties include Seebeck coefficients, electrical conductivity and thermal conductivity.

#### 3.4.1 Seebeck coefficient (*S*)

**Fig. 9:**
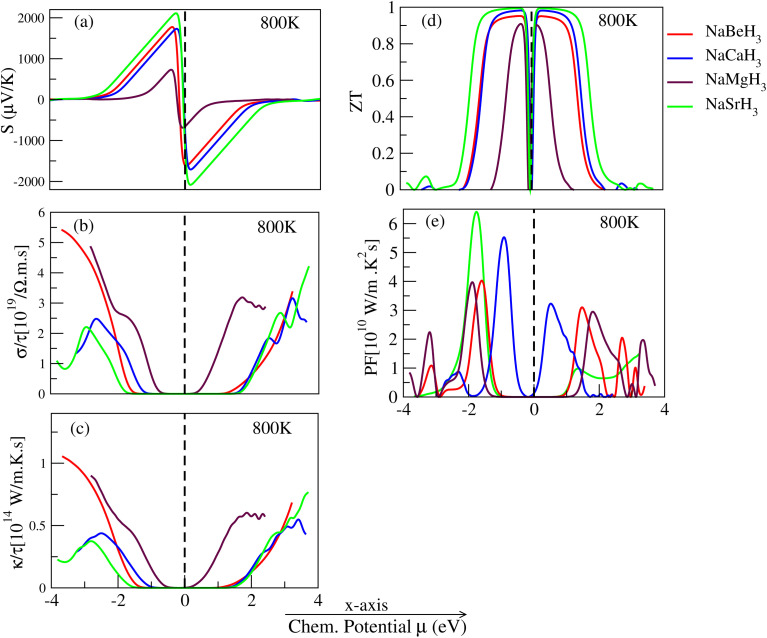
Thermoelectric properties of NaXH_3_ (X = Be, Mg, Ca, Sr).

How much voltage is transferred from a junction that is hotter to one that is colder is known as the thermo power. Varying temperature causes changes in the Seebeck coefficient. With rising temperatures, it gets smaller in the range 100 V K^−1^ to +1000 V K^−1^. Thermoelectric applications require substances with high Seebeck values. The Seebeck coefficient is the ability of a material to have a temperature gradient which produces a thermoelectric voltage. The Seebeck coefficient of NaBeH_3_, NaCaH_3_, NaSrH_3_ is presented in Fig. 9(a), at 800 K. The Seebeck coefficient values for NaBeH_3_, NaMgH_3_, NaCaH_3_, NaSrH_3_ are given in Table 3 and 4 for p-type and n-type doping, respectively. From Table 3 and 4 and Fig. 9(a), we show that NaSrH_3_ has the larger value at 800 K in n-type regions.

**Table 3:** Calculated values of Seebeck coefficient (*S*), electrical conductivity(*σ*), power factor (*P*), thermal conductivity (*K*) and figure of merit (*ZT*) in (p-type) region of thermoelectric property of NaBeH_3_, NaCaH_3_, NaMgH_3_, NaSrH_3_

Compound	p-type	*S* (V K^−1^)	*K* (W m^−1^ K^−1^ s^−1^)	*σ* (Ω^−1^ ms^−1^)	*P* (W mK^−2^ s^−1^)	*ZT*
NaBeH_3_	800 K	1700	0.61	3.4	2.9	0.945
NaCaH_3_	800 K	1730	0.54	3.1	3.2	0.977
NaMgH_3_	800 K	693	0.59	3.0	2.7	0.881
NaSrH_3_	800 K	2014	0.71	4.1	1.4	0.988

**Table 4:** Calculated values of Seebeck coefficient (*S*), electrical conductivity (*σ*), power factor (*P*), thermal conductivity (*K*) and figure of merit (*ZT*) in (n-type) region of thermoelectric property of NaBeH_3_, NaCaH_3_, NaMgH_3_, NaSrH_3_

Compound	n-type	*S* (V K^−1^)	*K* (W m^−1^ K^−1^ s^−1^)	*σ* (Ω^−1^ ms^−1^)	*P* (W m K^−2^ s^−1^)	*ZT*
NaBeH_3_	800 K	1757	1.04	5.3	4.0	0.951
NaCaH_3_	800 K	1790	0.44	2.5	5.4	0.972
NaMgH_3_	800 K	710	0.88	4.3	3.8	0.891
NaSrH_3_	800 K	2114	0.37	2.1	6.4	0.998

#### 3.4.2 Electrical conductivity (*σ*)

Electrical conductivity is a term for how a material can conduct electric currents. Using the strength of the electrical field to determine the current density ratio. While a thermoelectric material possesses a high electrical conductivity, the influence of joule heating should be minimum. Materials are categorized according on how well they conduct electricity; in contrast to insulators, conductors have a relatively high electrical conductivity. The Fermi level is nearer to the conduction band in a conductor than it is in a semiconductor, it is positioned in the middle of the valence band and conduction band. The Fermi level is nearer to the conduction band in p-type semiconductors than it is in n-type semiconductors, and *vice versa*. Temperature has a significant impact on electrical conductivity. Conductivity is 0 at absolute zero (K) temperatures, but it goes up exponentially as temperature goes up. The calculated electrical conductivities of NaBeH_3_, NaMgH_3_, NaCaH_3_, NaSrH_3_ are shown in Fig. 9(b) at 800 K and are listed in Table 3 and 4 at 800 K. The greatest electrical conductivity values are found for NaBeH_3_ at 800 K in the n-type region.

#### 3.4.3 Thermal conductivity (*κ*)

A material can conduct heat differently, and its ability to do so is called thermal conductivity. Heat means being warm or hot. Some materials do not let heat move very fast, while others let heat move quickly. Metals can usually let heat pass through them easily. Good conductors easily move heat while insulating materials do not – for instance, Styrofoam or rock wool. Materials that cannot transfer heat well are often used in things that need to stay cold and in things that stop heat from getting through. The calculated thermal conductivities of NaBeH_3_, NaMgH_3_, NaCaH_3_ and NaSrH_3_ are shown in Fig. 9(c) at 800 K and the calculated values of thermal conductivity of NaBeH_3_, NaMgH_3_, NaCaH_3_, NaSrH_3_ are presented in Table 3 and 4 at 800 K.

#### 3.4.4 Figure of merit (*ZT*)

The term “figure of merit” is a general concept used in various fields to quantify and compare the performance of a system or a device. The figure of merit is a number that shows how well the system works. In the particular study of materials that can conduct heat or convert it into electricity, scientists often use the figure of merit to measure how well a thermoelectric material will work. We use an equation to calculate it:4
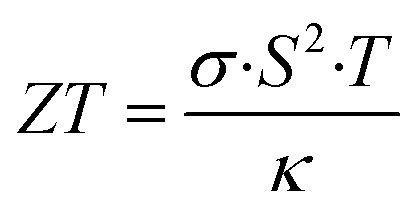
where *T* is the temperature; *κ* is the material's heat conductivity; *σ* is its electrical conductivity; *S* is its electrical generating capacity. To put it simply, a higher *ZT* value indicates that the material has superior heat-controlling capabilities, useful in appliances that run on electricity for heating or cooling. Fig. 9(d) illustrates the degree to which NaBeH_3_, NaMgH_3_, NaCaH_3_, NaSrH_3_ function at varying temperatures. Table 3 and 4 illustrate the observed values for NaXH_3_ (X = Be, Ca, Mg, Sr) for temperatures at 800 K. NaSrH_3_ has the maximum value in the n-type region, 0.998.

#### 3.4.5 Power factor (PF)

To calculate the thermoelectric power factor of a material, one of the best parameters to consider is the power factor (PF). PF = *σS*^2^, here ‘*S*’ represents the Seebeck coefficient and ‘*σ*’ represents the electrical conductivity of the material. The calculated power factor of NaBeH_3_, NaMgH_3_, NaCaH_3_, NaSrH_3_ are shown in Fig. 9(e) at 800 K, and the calculated power factor values are presented in Table 3 and 4. Further, it is shown in Fig. 9(e), that the n-type NaSrH_3_ has the largest value of PF making it favorable for thermoelectric applications. Among NaBeH_3_, NaCaH_3_, NaMgH_3_ and NaSrH_3_, the NaSrH_3_ is the perfect candidate for use in thermoelectric applications because it has the highest PF and high *ZT* in n-type doping.

To obtain a high *ZT* value, the material will have a large Seebeck coefficient, high electrical conductivity, and low thermal conductivity. It is evident from Table 3 and 4 that the value of Seebeck coefficient is close to unity. Therefore, all of these materials could be used in thermoelectric power generators.^45^ The thermoelectrical behavior of materials, including conductivity, energy band gaps, power factor and figure of merit, are essential for promoting the absorption and desorption of hydrogen. Positive thermoelectric characteristics may also help with heat control during the hydrogen cycle, which would boost storage efficiency even more. Furthermore, improving the interaction of electrical conductivity, thermal conductivity, and the Seebeck coefficient, can result in sophisticated materials that excel at both hydrogen storage and thermoelectric conversion. This integration promotes the development of sustainable energy solutions by increasing energy efficiency in hydrogen-based systems.

### 3.5 Elastic and mechanical properties

The strain-dependent matrix of second-order elastic constants (*C*_ij_), equilibrium volume and crystal energy are some of the parameters that affect a lattice's elastic behavior. The elastic stiffness tensor for NaXH_3_ (X = Be, Mg, Ca, Sr) compounds displaying the symmetry features of the *Pm*3̄*m* space group is composed of three separate components, denoted by Young’s notation as *C*_11_, *C*_12_, and *C*_44_.^46,47^ The calculated values of *C*_11_, *C*_12_ and *C*_44_ for NaXH_3_ (X = Be, Mg, Ca, Sr) perovskites are listed in Table 5. It is evident from the table that our calculated values of elastic constants fulfil the Born stability criteria, commonly known as the mechanical stability conditions.^48,49^

**Table 5:** Computed elastic constants (*C*_ij_) and Cauchy's pressure (*C*_p_) of NaXH_3_ (X = Be, Mg, Ca, Sr)

Compound	*C*_11_	*C*_12_	*C*_44_	*C*_p_
NaBeH_3_	84.738	35.037	61.473	−26.436
NaMgH_3_	119.26	16.062	0.1182	15.944
NaCaH_3_	76.264	1.326	12.816	−11.49
NaSrH_3_	75.222	2.759	12.707	−14.944

The positive/negative of Cauchy’s pressure (*C*_p_ = *C*_12_ − *C*_44_) can be used to evaluate whether a material exhibits ductile/brittle behavior. The data presented in the Table 5 indicate that NaXH_3_ (X= Be, Mg, Ca, Sr) display brittle behaviour, which is empirically determinable. The bulk modulus (*B*), Shear modulus (*G*), Young's modulus (*E*), Poisson's proportion (*v*) and Pugh’s ratio (*B*/*G*) are illustrations of the mechanical properties of the materials, calculated using the elastic constant (*C*_ij_), shown in Table 6, using equations given in ref. 50 and 51. The bulk modulus of a material indicates the maximum pressure it can bear without changing shape. The shear modulus shows how well a material can withstand pressure without losing its shape. The expansion and deformation of materials are due to their bulk and shear moduli. Young's modulus is a measure of a material's stiffness that compares how much it expands when pushed, to the amount of force applied to it. Table 6 shows that, in comparison to other materials, the material considered that has the largest bulk modulus *B* value is NaBeH_3_. The second column of results in the table indicate the shear *G* distortion, the compound with the greatest value for *G* was found to be NaBeH_3_. The values of *B* and *G* were used to calculate the young modulus *E*. In our calculations, a high Young's modulus *E* indicates that NaBeH_3_ is tougher than other materials.^52,53^ Materials that can bend a lot and do not break easily have a *B*/*G* ratio of more than 1.75, showing the ductile behaviour of the material. When Poisson's ratio gets close to 0.5, the material is likely to become difficult to compress. When *v* = 0.5, it is almost impossible to compress. Compared to other materials that can change shape easily, our estimated values for *v* range from 0.05 to 0.06, which shows that NaSrH_3_ is a material that is hard to compress. Table 6 provides the obtained values of anisotropic (*A*) for cubic NaXH_3_. The values of *A* significantly deviate from unity, indicating that these cubic materials, NaXH_3_, are anisotropic. Micro-hardness (*H*) is a measure of a material’s resistance to compression. According to the results in Table 6, NaBeH_3_ has a higher micro-hardness than other materials, which means it is more resistant to being compressed by small components.^54^

**Table 6:** Calculated bulk modulus (*B*), Shear modulus (*G*), Young’s modulus (*E*), Pugh's ratio (*B*/*G*), Poisson ratio (*v*), anisotropic index (*A*) and micro-hardness (*H*), of NaXH_3_ (X = Be, Mg, Ca, Sr)

Compound	*B* (GPa)	*G* (GPa)	*E* (GPa)	*B*/*G*	*v*	*A*	*H* (GPa)
NaBeH_3_	51.60	58.35	127.14	0.88	0.05	2.47	17.64
NaMgH_3_	50.46	17.35	46.72	2.90	0.23	0.02	3.40
NaCaH_3_	27.59	27.59	59.65	0.95	0.07	0.34	7.88
NaSrH_3_	26.91	32.06	68.84	0.83	0.06	0.48	9.60

## 4 Conclusion

In summary we considered the structural, formation energy, hydrogen storage, electronics, thermoelectric and elastic properties of NaXH_3_ (X = Be, Ca, Mg, and Sr) cubic hydride perovskites. The formation energy and elastic stability criteria confirm their mechanical stability. The band of the materials are increased by applying the mBJ correction. For NaXH_3_ (X = Be, Mg, Ca, and Sr), the computed hydrogen storage capacities are 8.6, 6.0, 4.6, and 2.6 wt%, respectively. Therefore, NaBeH_3_, NaMgH_3_, and NaCaH_3_ are considered to be potential materials for hydrogen storage applications. For NaSrH_3_, the power factor at negative chemical potentials is about 25–30% higher than positive chemical potentials, which indicates that the n-type doping is more efficient and the material could be suitable for thermoelectric applications.

## Data availability

The authors confirm that the data supporting the findings of this study are available within the article.

## Conflicts of interest

There are no conflicts to declare.
